# New karyotype for *Mesomys stimulax* (Rodentia, Echimyidae) from the Brazilian Amazon: A case for species complex?

**DOI:** 10.1002/ece3.7583

**Published:** 2021-05-08

**Authors:** Stella Miranda Malcher, Julio Cesar Pieczarka, Adenilson Leão Pereira, Paulo José Siqueira do Amaral, Rogério Vieira Rossi, Juliane Saldanha, Cleusa Yoshiko Nagamachi

**Affiliations:** ^1^ Laboratório de Citogenética Centro de Estudos Avançados da Biodiversidade Instituto de Ciências Biológicas Universidade Federal do Pará Belém Brasil; ^2^ Faculdade de Medicina Universidade Federal do Pará Altamira Brasil; ^3^ Centro Universitário do Estado do Pará Belém Brasil; ^4^ Laboratório de Mastozoologia Instituto de Biociências Universidade Federal do Mato Grosso Cuiabá Brasil

**Keywords:** chromosomal differences, cytogenetics, Eumysopinae, FISH, Pará spiny tree rat

## Abstract

*Mesomys* Wagner, 1845 (Rodentia, Echimyidae, Eumysopinae) currently has four recognized species, three of which occur in Brazil: *Mesomys hispidus* (probably a species complex), *M*. *occultus,* and *M*. *stimulax*. *Mesomys leniceps* is found in montane forests of northern Peru. *Mesomys stimulax*, the focus of the present study, has a distribution that is restricted to the central and eastern Amazonia south of the Amazon River, extending from the left bank of the Tapajós River to the right bank of the Tocantins River, and south to the southeast portion of Pará State. The genus presents karyotypes with diploid number 2n = 60 and Fundamental Number (FN) = 116 for *M*. *hispidus* and *M*. *stimulax*, and 2n = 42, FN = 54 for *M*. *occultus*. We studied the karyotype of a female specimen of *M*. *stimulax* collected from the Tapirapé‐Aquiri National Forest, Marabá, Pará, Brazil, in the Xingu/Tocantins interfluvium. The obtained karyotype (2n = 60 and FN = 110) differs from that described in the literature for both *M*. *stimulax* and *M*. *hispidus* by exhibiting more biarmed chromosomes, probably due to pericentric inversions and/or centromeric repositioning, and exhibiting differences in the amount and distribution of constitutive heterochromatin (CH). These results suggest that, similar to what has already been proposed for *M*. *hispidus*, *M*. *stimulax* may represent a species complex and/or cryptic species. The mechanisms of chromosomal diversification in *Mesomys* and the biogeographic implications are discussed reinforcing the need for broad systematic review for *Mesomys*.

## Graphical abstract

We studied the karyotype of *Mesomys stimulax* collected from the Amazon rainforest. The obtained karyotype (2n = 60 and FN = 110) differs from that described in the literature. These results suggest that *M*. *stimulax* may represent a complex of species and/or cryptic species.

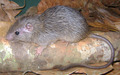

## 1 INTRODUCTION

Rodents of genus *Mesomys* Wagner, 1845 (Echimyidae, Eumysopinae) are generally uncommon components of the Amazonian fauna, inhabiting primary and secondary forests (Emmons & Feer, 1997). They are arboreal, nocturnal, solitary, and difficult to capture; as such, they have little representation in scientific collections and are largely unstudied (Patton & Emmons, 2015).

**FIGURE 1:**
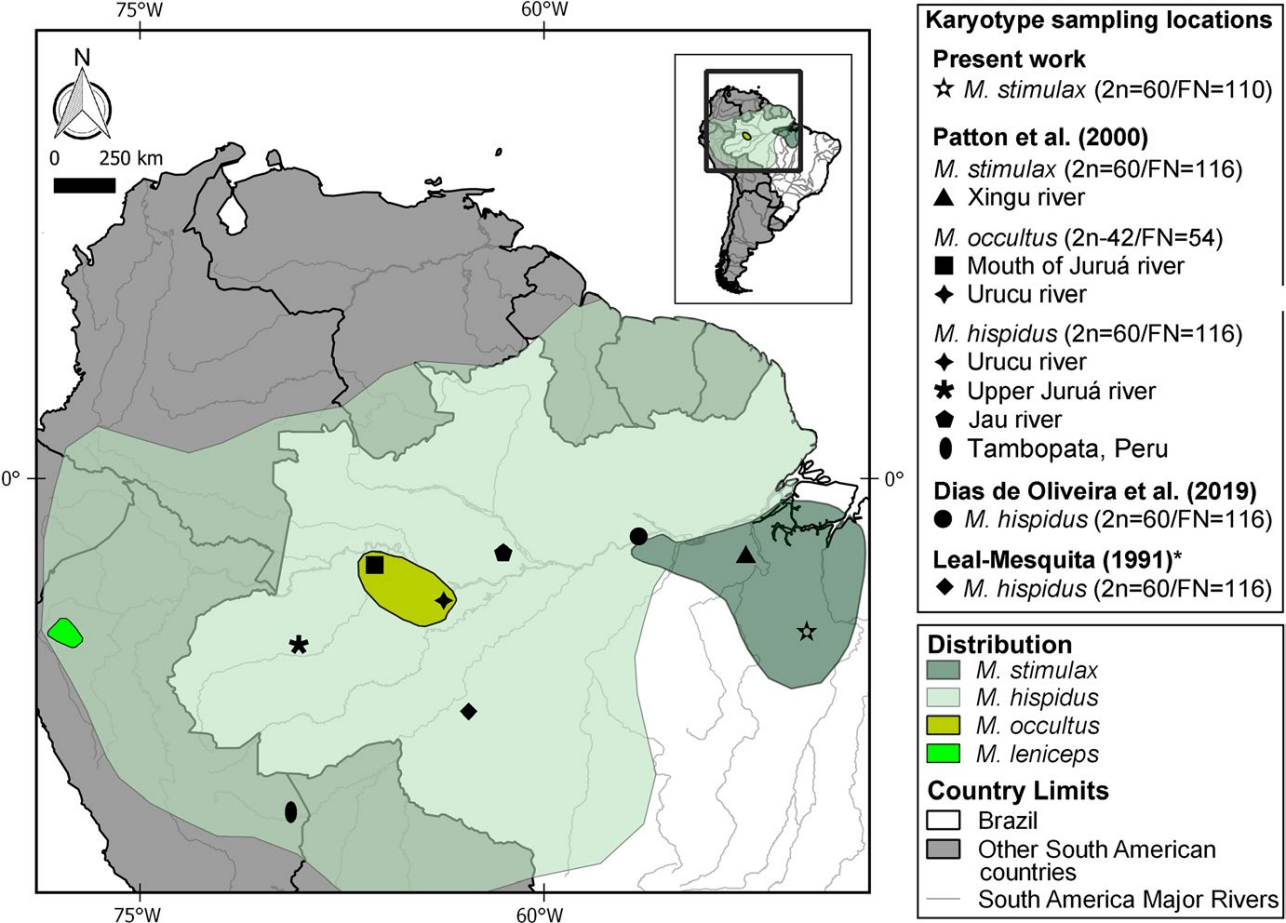
Map showing the distribution area of *Mesomys* species (Patton & Emmons, 2015) with highlights indicating the collection sites for karyotyped samples described in the literature and the present work. The map was made using QUANTUM‐GIS (Q‐GIS) v. 3.8.0 by Willam Oliveira da Silva. The database was obtained from DIVA and REDLIST. Scale bar: 5 cm

Currently, four species are recognized for the genus: *Mesomys hispidus* (Desmarest, 1817), the type species (which includes *M*. *ecaudatus* Wagner, 1845, *Echimys* *ferrugineus* Günther, 1876, and *M*. *ferrugineus* *spicatus* Thomas, 1924 as synonyms); *Mesomys stimulax* Thomas, 1911; *Mesomys leniceps* Thomas, 1926; and *Mesomys occultus* Patton, da Silva and Malcolm, 2000 (Patton et al., 2000; Patton & Emmons, 2015; Woods & Kilpatrick, 2005). Of them, *M*. *leniceps* is the unique species not reported for Brazil, being restricted to northern Peru; *Mesomys hispidus* is the most widely distributed and is present throughout nearly all of the Amazonia; *Mesomys occultus* occurs on the left bank of the Juruá River, south of the Solimões River and Rio Urucú, Tefé, Amazonas, Brazil (Patton et al., 2000); and *Mesomys stimulax* is restricted to central and eastern Amazonia, south of the Amazon River, with a distribution that extends from the left bank of the lower/medium Tapajós River to the right bank of the Tocantins River, and south to the southeastern region of Pará State (Miranda & Silva, 2015; Patton & Emmons, 2015; Figure 1). Molecular approaches indicate that the species diversity of *Mesomys* is underestimated, with *M*. *hispidus* likely representing a species complex (Orlando et al., 2003; Patton et al., 2000).

The basic karyotypes from three of the four species of the genus have been described. The karyotype 2n = 42 and FN = 54 was reported for individuals of *M*. *occultus* collected from the region of the Juruá River (Patton et al., 2000). The karyotype 2n = 60 and FN = 116 was described for individuals of *M*. *hispidus* collected from sites at the Samuel Dam in Madeira River (Leal‐Mesquita, 1991), the Juruá River south of the Solimões River, the upper Urucu River, Jaú River north of the Solimões River, Brazil, and Tambopata, Peru (Patton et al., 2000; Emmons, personal communication). This same karyotype (2n = 60 and FN = 116) was assigned to specimens of *M*. *stimulax* collected from both banks of the lower Xingu River (Patton et al., 2000; Emmons, personal communication) and from the left bank of the lower Tapajós River (Dias de Oliveira et al., 2019). Only the *M*. *stimulax* karyotype (2n = 60; FN = 116) has been analyzed with chromosome banding and molecular cytogenetics (Dias de Oliveira et al., 2019). More detailed cytogenetic studies of these species are needed to improve our understanding of the real karyotypic diversity in this genus and shed light on the mechanisms involved in its diversification.

In the present study, we report a new karyotype for *M*. *stimulax*, from an individual collected in Tapirapé‐Aquiri National Forest, Marabá, Pará, Brazil. The mechanisms of chromosomal diversification, the biogeographic implications, and the possibility of cryptic speciation are discussed.

## 2 MATERIALS AND METHODS

### 2.1 Sample

**FIGURE 2:**
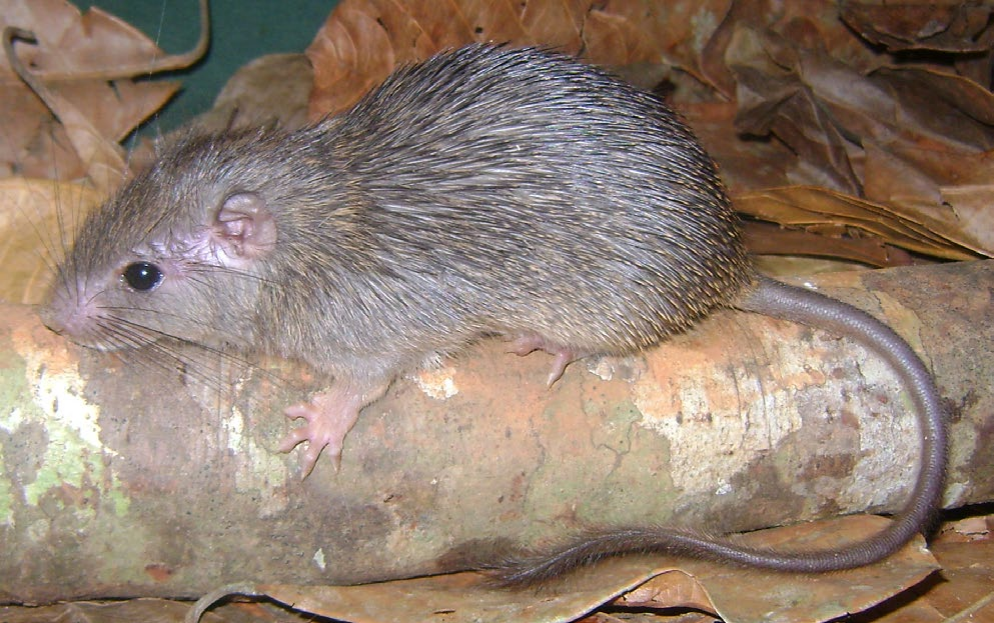
A female specimen of *Mesomys stimulax* studied herein, deposited at the Museu Paraense Emilio Goeldi (MPEG 42030) in Belém, Pará, Brazil. Image courtesy of Cleuton Lima Miranda

The sample consisted of a female specimen of *Mesomys* (Figure 2), which was collected using a live animal trap (Sherman) baited with a mixture of peanut butter, sardine, and cornflour, set in the understory (ca. 1.5 m above the ground) at Igarapé Mano, Tapirapé‐Aquiri National Forest, Marabá, Pará (05°46′21″S, 110 50°33′21″W, Figure 1), in the Xingu‐Tocantins interfluvium. This was the only specimen of *Mesomys* collected in four field expeditions, during which a total effort of 16,150 trap‐nights of Sherman and wire cage traps and 4,800 bucket‐days of pitfall traps were employed. JCP has a permanent field license, number 13248, from the “Chico Mendes Institute for Biodiversity Conservation”. The CEABIO (Centro de Estudos Avançados da Biodiversidade) Cytogenetics Laboratory at Universidade Federal do Pará, Belém, Pará, Brazil, has authorization number 19/2003 from the Ministry of the Environment for the transportation of samples, as well as for the use of samples for research under number 52/2003. This research was approved by the Ethics Committee of the Federal University of Pará (Permission 68/2015). The specimen has been deposited at the Museu Paraense Emilio Goeldi (MPEG 42030) in Belém, Pará, Brazil.

### 2.2 Identification procedures

The specimen was identified by morphological analysis following Patton and Emmons (2015) and Miranda and Silva (2015). In addition, tissue sample was used to extract DNA and obtain a partial sequence of the mitochondrial gene cytochrome b (cyt b) used in a phylogenetic analysis, as follows. Extraction, amplification, and sequencing of Cytb protocols followed Saldanha et al. (2019). The data matrix was complemented with sequences of nine specimens from the GenBank, including one representative of *M*. *occultus*, one representative of each clade of *M*. *hispidus* recognized by Orlando et al. (2003), and all available specimens of *M*. *stimulax* (Table S1). Sequences of *M*. *leniceps* and representatives of clades B, E, and F recognized by Orlando et al. (2003) are not available in the GenBank. The sequences were aligned and edited in the program BioEdit 7.0.5.2 (Hall, 1999). The data matrix was best represented by the *Transition model* with invariable proportion of sites and gamma distribution (TIM2 + I + G) generated by the program JModeltest2 (Darriba et al., 2012). On CIPRES platform (Miller et al., 2010), a Bayesian inference analysis was performed through the MrBayes 3.2.7a program (Ronquist et al., 2012) with four chains, 50 million generations, a sampling tree each 1,000 generation, and 25% burn‐in. The trees obtained were visualized and edited in the FigTree program v1.4.3 (Rambaut, 2016), and branch supports were evaluated by Bayesian posterior probability. The genetic distances among clades were calculated in the MEGA7 (Kumar et al., 2016) by uncorrected p‐distance method. The species *Lonchothrix emiliae* Thomas, 1920 was used as outgroup based on broad phylogenetic studies with the Echimyidae family (Courcelle et al., 2019; Fabre et al., 2016; Upham et al., 2013).

### 2.3 Cytogenetic analysis

Chromosomal preparations were obtained from the bone marrow in the field (Ford & Harmerton, 1956). As our sample is a female, the definition of the X chromosome was made by comparing it with the literature. The following techniques were applied, with adaptations: G‐banding (Sumner et al., 1971), C‐banding (Sumner, 1972), Ag‐NOR staining (Howell & Black, 1980), and FISH (Fluorescence In Situ Hybridization) with telomeric probes (All Human Telomere Probe: Oncor, P5091) (Nagamachi et al., 2013) and 18S rDNA probes (Hatanaka & Galetti, 2004). Images of classic cytogenetics were obtained using an Olympus BX41 microscope (bright field/phase) with a digital CCD 1300QDS camera and analyzed using the SpectraView software (Applied Spectral Imaging). Images of FISH were obtained using a Nikon H550S microscope and analyzed using Nis‐Elements software. The images were edited using the Adobe Photoshop CS4 program.

## 3 RESULTS

### 3.1 Morphological and molecular identification

The specimen karyotyped in this study is a nonadult individual in age class 5 according to age criteria provided by Patton and Rogers (1983) and Leite (2003) for echimyid rodents, molting to the adult pelage. The adult part of its pelage agrees with *M*. *stimulax* descriptions provided by Miranda and Silva (2015) and Patton and Emmons (2015), such as dorsal pelage strongly washed with orange, subterminal band present on the aristiform hairs of neck and shoulders, and ventral pelage cream, with white throat, axillae, and inguinal regions. The specimen has 136 mm of head and body length, 145 mm of tail length, and 81 g of body mass. Because of its early age, external and craniodental measurements are not useful for identification purposes.

Our phylogenetic analysis (Figure S1) recovered *Mesomys* as monophyletic, with *M*. *occultus* as the sister species of *M*. *hispidus* + *M*. *stimulax*, from which it differed by 11.44% of mean genetic distance. The species *M*. *hispidus* and *M*. *stimulax* were recovered as monophyletic groups, with respectively 6.27% and 3.38% of mean intraclade genetic distances, and 7.68% of mean genetic distance between them. The specimen karyotyped and sequenced in this study (voucher museum number MPEG 42030) was recovered as sister to other two specimens of *M*. *stimulax* from the east bank of Xingu river (voucher field numbers LHE 572 and MDC 550; Patton et al., 2000), from which it differed by 2.38% of mean genetic distance. Two specimens identified as *M*. *stimulax* in the GenBank (LTJ 65 and RMNH.MAM.21728) were nested with specimens of *M*. *hispidus*.

### 3.2 Karyotype

**FIGURE 3:**
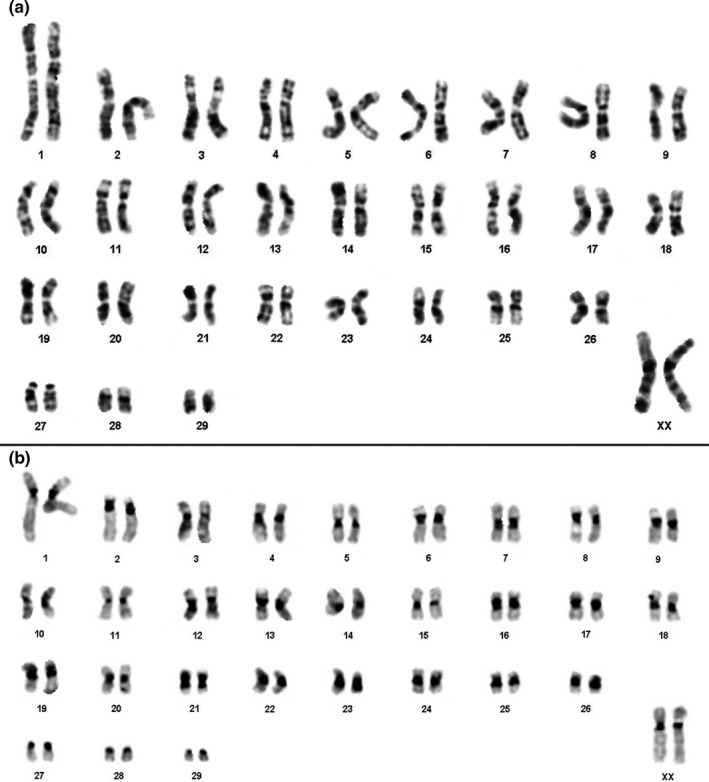
Karyotype of *Mesomys stimulax* with 2n = 60 and FN = 110: (a) G‐banding G and (b) C‐banding

The specimen of *Mesomys stimulax* studied herein has 2n = 60 and FN = 110, with 26 pairs of biarmed chromosomes, three small acrocentric pairs, and a medium‐size submetacentric X chromosome (Figure 3a; Figure S2). Constitutive Heterochromatin (CH, Figure 3b) occurs in large blocks in the centromeric regions of all pairs and in the pericentromeric regions of most chromosomes, with the exception of pairs 11, 15, 27, 28, and 29. Some chromosomes are almost entirely heterochromatic (pairs 21, 23, and 26).

**FIGURE 4:**
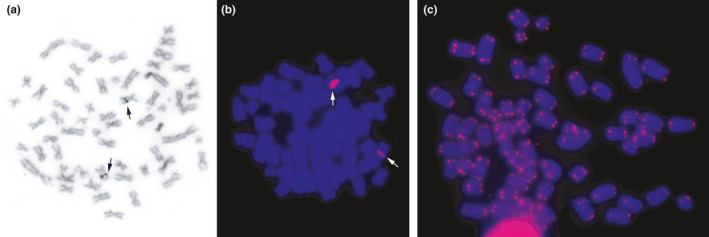
*Mesomys stimulax* metaphases with 2n = 60 and FN = 110. (a) Nucleolar Organizer Regions (NOR) staining; (b) FISH with 18S rDNA probes; and (c) FISH with telomeric probes

Ag‐NO3 staining shows that the Nucleus Organizing Region (NOR) in *M*. *stimulax* occurs in the interstitial region of long arm of pair 8 (Figure 4a). This staining coincides with hybridization of 18S rDNA probes on FISH (Figure 4b). FISH with human telomeric sequences shows hybridization at distal portions of all chromosomal pairs, with no Interstitial Telomeric Sequences (ITS) observed (Figure 4c).

## 4 DISCUSSION

Both the morphological and phylogenetic analyses carried out in the present study allowed to characterize our sample as *M*. *stimulax*. The phylogenetic analysis (Table S1 and Figure S1) also showed that two samples from the GenBank must be reidentified. The specimen LTJ 65 karyotyped by Dias de Oliveira et al. (2019) is actually *M*. *hispidus* and not *M*. *stimulax* as those authors supposed. The mistake was due to the small number of sequences analyzed in the phylogeny of that study and the misidentification of the specimen RMNH.MAM.21728 as *M*. *stimulax* by Fabre et al. (2016), whose sequence was recovered as sister to Dias de Oliveira's et al. (2019) karyotyped specimen.

The karyotype of *M*. *stimulax* described herein (2n = 60, FN = 110) differs from that previously described for this species (2n = 60 and FN = 116; Patton et al., 2000; Emmons, personal communication) in the number of autosomal arms (FN) due to our specimen MPEG 42030 carries three acrocentric pairs (pairs 27, 28, and 29; Figure 3), while the previously reported karyotype had only biarmed chromosomes.

*Mesomys hispidus* (Dias de Oliveira et al., 2019; Orlando et al., 2003; Patton et al., 2000) shares the same 2n and FN of the previously reported specimens of *M*. *stimulax* (Patton et al., 2000) and probably shares the same chromosomal differences with the karyotype of *M*. *stimulax* herein described. In our sample, more CH was present in the centromeric and pericentromeric regions of almost all pairs, with some large blocks observed in pairs 21, 23, and 26 (Figure 3b) while in *M*. *hispidus* there are no such large blocks (Dias de Oliveira et al., 2019). The difference in FN may be due to pericentric inversions or centromeric repositioning (Rocchi et al., 2012), while the variation in amount of CH is likely to reflect the addition/deletion of repetitive sequences.

This 2n = 60 and FN = 116 karyotype is also found in the *Isothrix bistriata* (Patton et al., 2000), whose genus is a sister group of the clade formed by *Mesomys* and *Lonchothrix* (e.g., Emmons & Fabre, 2018; Fabre et al., 2016). These data suggest that this is the ancestral karyotype of the clade formed by *Isothrix* and *Mesomys*. Therefore, the karyotype described in the present study (2n = 60, FN = 110) must be derived from the most common karyotype in the genus. This interpretation also makes sense from a geographic point of view. The karyotype of *M*. *stimulax* collected near Altamira on both banks of the lower Xingu River (Patton et al., 2000; Emmons, personal communication) is similar to the possible ancestral one (2n = 60, FN = 116). This locality is close to the distribution area of *M*. *hispidus* which has the same karyotype (also 2n = 60, FN = 116; Dias de Oliveira et al., 2019). Our sample has a derived karyotype (2n = 60, FN = 110) and it was collected some 500 km southeast, suggesting that *M*. *stimulax* expanded its geographic distribution from the west to the southeast on the Amazon region.

As large Amazonian rivers can act as primary or even secondary geographic barriers for rodents (e.g., Antonelli et al., 2018; Leite & Rogers, 2013; Oliveira da Silva et al., 2017; Patton et al., 2000; Patton & Emmons, 2015), the biota of the Amazon region may have a complex evolutionary history (Antonelli et al., 2018). However, one of the clades of *M*. *hispidus* is believed to have crossed the Amazon River (or the original population was divided by the Amazon River), as it was distributed from the Guiana Shield to the Bolivian Chaco (Orlando et al., 2003). Thus, it is not clear whether rivers are effective barriers for *Mesomys*. The existence of *Mesomys* with the same karyotype on both banks of the Xingu River suggests that this river may not be a strong barrier for this genus. As this is the karyotype that we supposed to be the ancestral for *Mesomys*, an alternative possibility is that this distribution is consequence of its ancestral condition.

The existence of two different cytotypes for *M*. *stimulax* (FN = 116 mentioned by Patton et al., 2000; FN = 110 here described) collected from different locations (Figure 1) led us to question whether *M*. *stimulax* constitutes a species complex or a single species with karyotypic variants. Different karyotypes in morphologically indistinguishable species (cryptic species) are quite frequent in rodents, as previously described for *Proechimys* (Eler et al., 2020; Rodrigues da Costa et al., 2016), *Neacomys* (Oliveira da Silva et al., 2017, 2019), and *Oecomys* (Malcher et al., 2017). Morphological and molecular studies in specimens of *M*. *hispidus* from different locations suggest that this taxon must comprise more than one species (Orlando et al., 2003). The situation may be similar for *M*. *stimulax*. In our phylogenetic analysis (Figure S1), we found a genetic distance of 4.78% between the *M*. *stimulax* sequence of Upham and Patterson (2015) and the other samples, which supports the possibility that *M*. *stimulax* is a species complex. Cytogenetic studies of more samples of *M*. *stimulax* are needed to define the existence and territorial extent of a possible population with 2n = 60, FN = 110. Moreover, molecular and morphological studies will be useful to properly evaluate and describe the nature of the diversity found in this still largely unstudied group of rodents.

## 5 CONCLUSION

The species *M*. *stimulax* shows variation in its karyotypic formula, suggesting that this name may refer to more than one species. This could have originated from populations in the western region whose distribution expanded from the west to the east of the Amazon. Our results emphasize the need for a systematic and biogeographic study with more samples and an integrative approach.

## CONFLICT OF INTEREST

The authors declare no conflict of interest.

## AUTHOR CONTRIBUTIONS

**Stella Miranda Malcher:** Conceptualization (equal); data curation (equal); formal analysis (equal); investigation (equal); methodology (equal); visualization (equal); writing‐original draft (equal); writing‐review & editing (equal). **Julio Cesar** **Pieczarka:** Formal analysis (equal); funding acquisition (equal); methodology (equal); resources (equal); visualization (equal); writing‐review & editing (equal). **Adenilson Leão Pereira:** Methodology (equal); writing‐review & editing (equal). **Paulo José** **Siqueira do Amaral:** Methodology (equal); writing‐review & editing (equal). **Rogério Vieira Rossi:** Data curation (equal); formal analysis (equal); investigation (equal); methodology (equal); resources (equal); writing‐review & editing (equal). **Juliane Saldanha:** Formal analysis (equal); methodology (equal); writing‐review & editing (equal). **Cleusa Yoshiko Nagamachi:** Formal analysis (equal); funding acquisition (equal); methodology (equal); project administration (equal); resources (equal); supervision (equal); visualization (equal); writing‐review & editing (equal).

## Supporting information

Fig S1

Fig S2

Table S1

Supplementary Material

## Data Availability

All data used in this research are available in the article. There was no need to deposit in public databases. The authors are available for any further explanation.
