# Supplementary figures and images for: New karyotype for Mesomys stimulax (Rodentia, Echimyidae) from the Brazilian Amazon: A case for species complex?
Source: Ecol Evol. 2021 May 8;11(12):7125–31. doi: 10.1002/ece3.7583 (PMC8216883; doi:10.1002/ece3.7583)

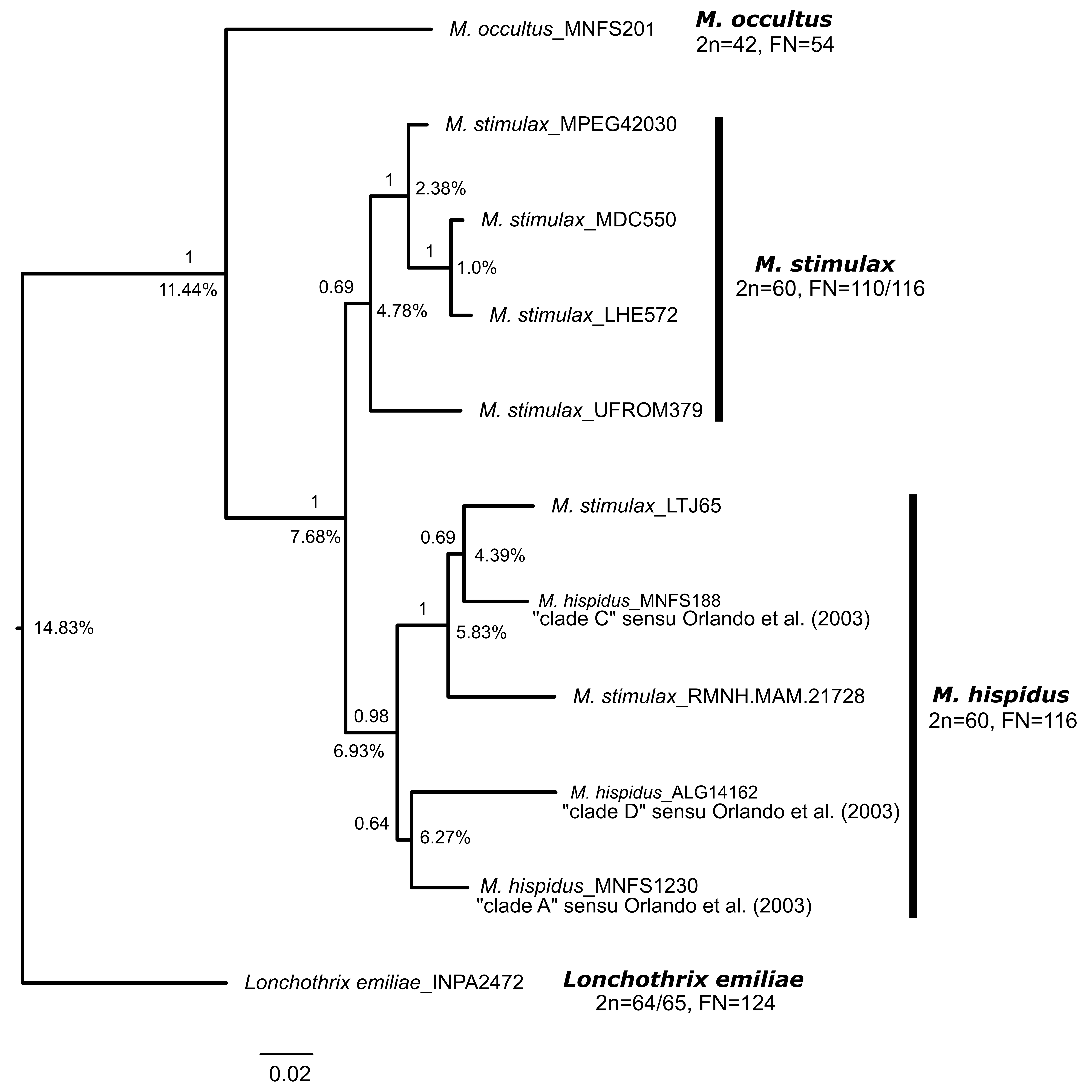

Supplement: Supplementary file 1 — Fig S1 [file ECE3-11-7125-s004.jpg]

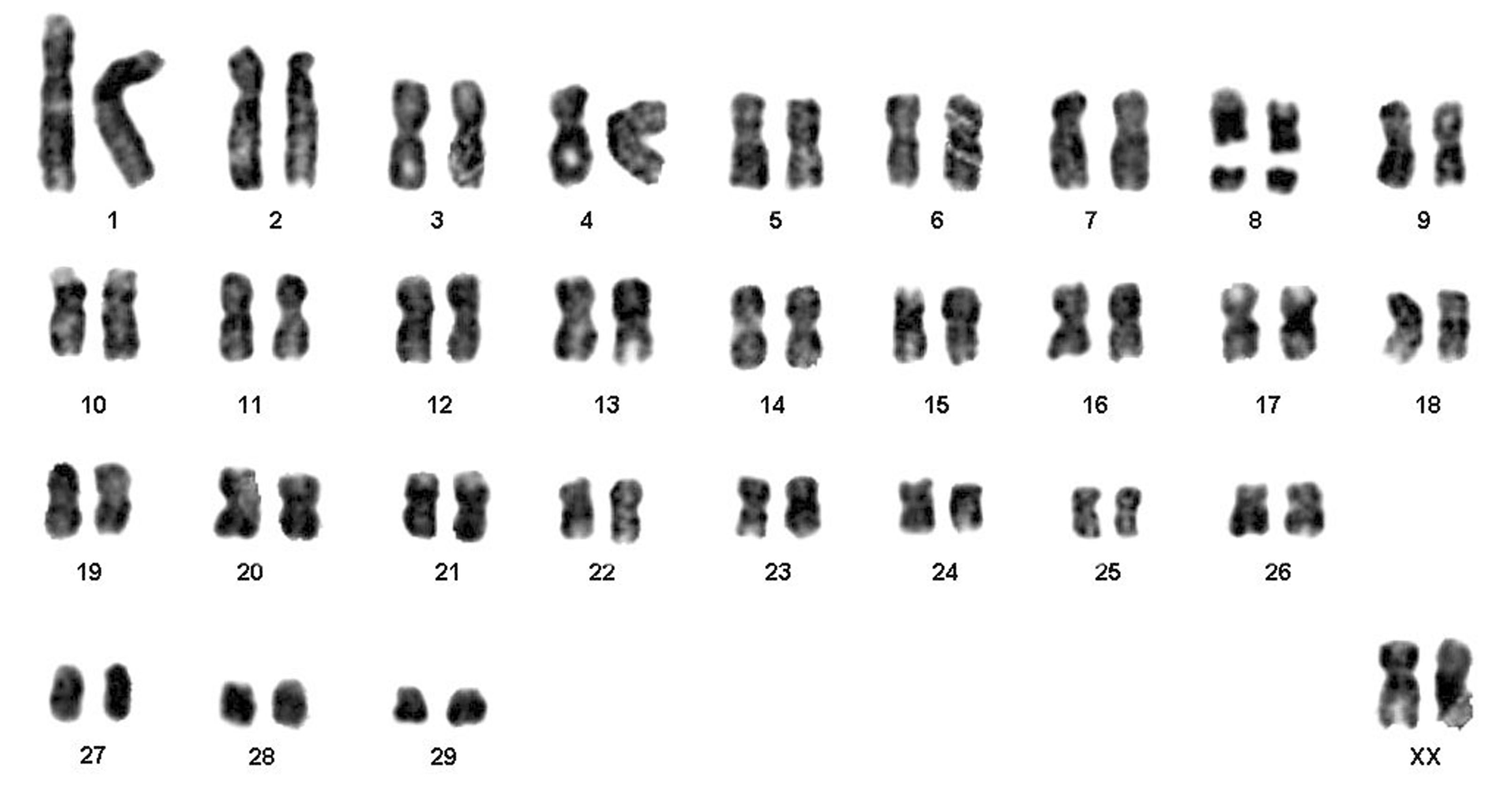

Supplement: Supplementary file 2 — Fig S2 [file ECE3-11-7125-s001.jpg]
