# Supplementary material for: New karyotype for Mesomys stimulax (Rodentia, Echimyidae) from the Brazilian Amazon: A case for species complex?
Source: Ecol Evol. 2021 May 8;11(12):7125–31. doi: 10.1002/ece3.7583 (PMC8216883; doi:10.1002/ece3.7583)
Supplement: Supplementary file 3 — Table S1 [file ECE3-11-7125-s003.docx]

| **Collection number** | **Field number** | **Genbank access number** | **Species/clade** | **Reference** |
| --- | --- | --- | --- | --- |
| USNM 549808 | LHE 572 | L23392 | *M. stimulax* | Silva & Patton (1993) |
| MPEG 28606 | MNFS 1230 | L23366 | *M. hispidus* “clade A” sensu Orlando et al. (2003) | Silva & Patton (1993) |
|  | MNFS 188 | L23379 | *M. hispidus* “clade C” sensu Orlando et al. (2003) | Silva & Patton (1993) |
|  | ALG 14162 | L23371 | *M. hispidus* “clade D” sensu Orlando et al. (2003) | Silva & Patton (1993) |
|  | MNFS 201 | L23382 | *M. occultus* | Silva & Patton (1993) |
|  | UFROM 379 | KJ742667 | *M. stimulax* | Upham & Patterson (2015) |
| USNM 549807 | MDC 550 | L23389 | *M. stimulax* | Silva & Patton (1993) |
| RMNH.MAM.21728 |  | KU892788 | *M. stimulax* | Fabre et al. (2016) |
|  | LTJ 65 | MW822549 | *M. hispidus* * | Dias de Oliveira et al. (2019) |
| MPEG 42030 | PSA 188 | MW807421 | *M. stimulax* | Present study |
| INPA 2472 |  | AF422921 | *Lonchothrix emiliae* | Leite & Patton (2002) |

* Published by Dias de Oliveira as *M. stimulax*

Dias de Oliveira, L., Oliveira da Silva, W., Rodrigues da Costa, M.J., Sampaio, I., Pieczarka, J.C., & Nagamachi, C.Y. (2019). First cytogenetic information for *Lonchothrix emiliae* and taxonomic implications for the genus taxa *Lonchothrix* + *Mesomys* (Rodentia, Echimyidae, Eumysopinae). PLoS ONE, 14(4): e0215239. https://doi.org/10.1371/journal.pone.0215239

Fabre, P.H., Upham, N.S., Emmons, L.H., Justy, F., Leite, Y.L., Loss, A.C., Orlando, L., Tilak, M.K., Patterson, B.D., & Douzery, E.J. (2016). Mitogenomic phylogeny, diversification, and biogeography of South American spiny rats. Molecular Biology and Evolution, 34 (3): 613-633.

Leite,Y.L. & Patton,J.L. (2002). Evolution of South American spiny rats (Rodentia, Echimyidae): the star-phylogeny hypothesis revisited. Molecular Phylogenetics and Evolution, 25 (3): 455-464.

Silva, M.N.F. & Patton, J.L. (1993). Amazonian phylogeography: mtDNA sequence variation in arboreal. Molecular Phylogenetics and Evolution, 2: 243-255.

Upham,N.S. & Patterson,B.D. (2015). Phylogeny and evolution of caviomorph rodents (Rodentia, Hystricomorpha): a complete timetree for living genera. Pp. 63-120 in Vassallo, A.I., Antenucci, D. (editors). Biology of caviomorph rodents: diversity and evolution. SAREM Series A, Buenos Aires.
