# Supplementary material for: New karyotype for Mesomys stimulax (Rodentia, Echimyidae) from the Brazilian Amazon: A case for species complex?
Source: Ecol Evol. 2021 May 8;11(12):7125–31. doi: 10.1002/ece3.7583 (PMC8216883; doi:10.1002/ece3.7583)
Supplement: Supplementary file 4 — Supplementary Material [file ECE3-11-7125-s002.docx]

Supplementary Material

**Table S1.** Specimens employed in the molecular phylogenetic analysis.

**Figure S1.** Bayesian Inference topology of selected *Mesomys* specimens based on mitochondrial cyt b gene sequences with 798 base pairs. Numbers above branches indicate Bayesian posterior probabilities, and percentage values indicate mean genetic distances between clades calculated by uncorrected p-distance method. The sample data are provided in Supplementary Material, Table 1. Karyotype information is provided for the species.

**Figure S2.** A conventional staining karyotype of *Mesomys stimulax* studied herein (2n = 60 and FN = 110).
